# Cutaneous Manifestations and Dermatologic Adverse Events in IBD: A Clinical Update

**DOI:** 10.1093/ibd/izaf228

**Published:** 2025-10-23

**Authors:** Dimitrios Tsilimpotis, Georgia Kyriakou, Luc Biedermann, Stephan Vavricka, Thomas Greuter, Gerhard Rogler, Michael Doulberis

**Affiliations:** Department of Dermatology, Medical School, University of Patras, 26504 Patras, Greece; Department of Dermatology, Medical School, University of Patras, 26504 Patras, Greece; Department of Gastroenterology and Hepatology, University Hospital Zurich, University of Zurich, 8091 Zurich, Switzerland; Department of Gastroenterology and Hepatology, University Hospital Zurich, University of Zurich, 8091 Zurich, Switzerland; Zentrum für Gastroenterologie und Hepatologie, 8002 Zurich, Switzerland; Department of Gastroenterology and Hepatology, University Hospital Zurich, University of Zurich, 8091 Zurich, Switzerland; Department of Gastroenterology and Hepatology, University Hospital Lausanne, 1011 Lausanne, Switzerland; Department of Gastroenterology and Hepatology, GZO-Zurich Regional Health Center, 8620 Wetzikon, Switzerland; Department of Gastroenterology and Hepatology, University Hospital Zurich, University of Zurich, 8091 Zurich, Switzerland; Department of Gastroenterology and Hepatology, University Hospital Zurich, University of Zurich, 8091 Zurich, Switzerland; Division of Gastroenterology and Hepatology, Medical University Department, Kantonsspital Aarau, 5001 Aarau, Switzerland

**Keywords:** IBD, dermatological manifestations, biologics, extraintestinal, Crohn, ulcerative colitis

## Abstract

Inflammatory bowel diseases (IBDs), including Crohn’s disease and ulcerative colitis, are immune-mediated disorders characterized by chronic gastrointestinal inflammation and a broad spectrum of extraintestinal manifestations. Among these, dermatological manifestations significantly impact patients’ quality of life (QoL), presenting as conditions linked to IBD itself or IBD-related medical therapy. This comprehensive review underscores the relationship between IBD and cutaneous manifestations, with particular emphasis on erythema nodosum, pyoderma gangrenosum, Sweet syndrome, and hidradenitis suppurativa, alongside autoimmune conditions like psoriasis and vitiligo. The influence of biologic therapies, including both paradoxical skin reactions mimicking extraintestinal manifestations and nonspecific rashes, is also discussed, with a focus on the pathophysiological mechanisms and therapeutic approaches. Emerging evidence highlights the bidirectional interplay of gut-skin axis, with shared genetic, microbial, and immune pathways. Special considerations, such as pregnancy-related dermatoses, are included to provide a holistic view of this complex relationship. Improved comprehension of these manifestations not only emphasizes the necessity for interdisciplinary care, but also informs tailored therapies to address systemic inflammation while minimizing dermatological complications. This update offers practical insights and emerging evidence to guide clinicians in optimizing patient outcomes.

## Graphical abstract

**Graphical Abstract:**
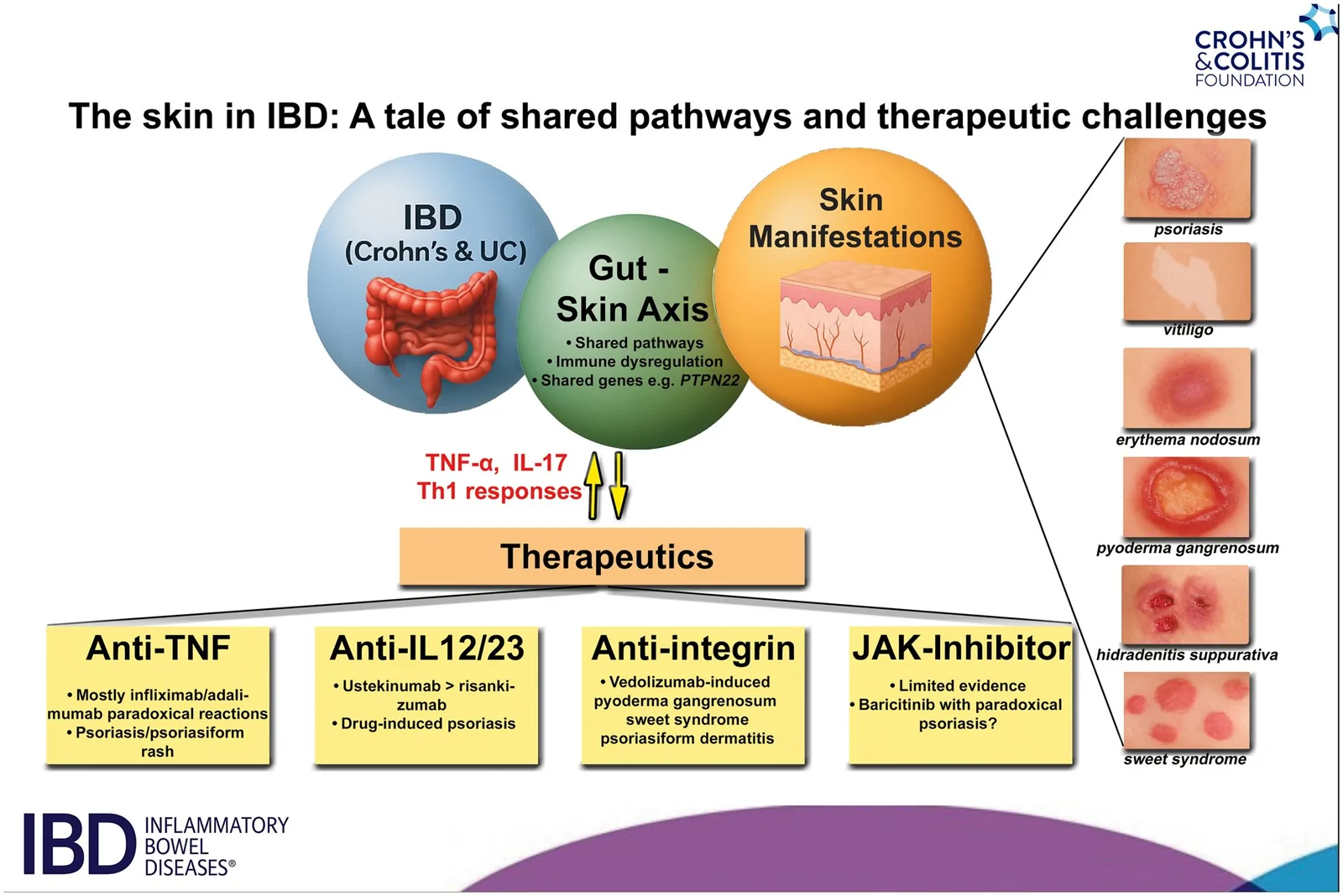

## Introduction

Inflammatory bowel disease (IBD) is an umbrella term that refers to immune-mediated diseases characterized by chronic, relapsing-remitting gastrointestinal tract inflammation. The main symptoms include diarrhea, abdominal pain, fever, presence of blood in stools (especially in ulcerative colitis) and weight loss, which variably parallel the disease course.^1^^,^^2^

The two most important forms of IBD include ulcerative colitis (UC) and Crohn’s disease (CD). UC is a nontransmural mucosal inflammation restricted to the colon, albeit backwash ileitis is an acknowledged less frequent (<10%) feature of it.^2^^,^^3^ CD, on the other hand, is characterized by transmural inflammation as well as by skip lesions that can affect any part of the gastrointestinal tract, from the mouth to the anus.^2^ However, the most common localization is the ileocecal region. Due to the transmural nature of inflammation, fistulas and strictures are common hallmarks of the latter disease. Clinical presentation depends largely on disease location, severity of inflammation, and accompanied complications.^2^ A less common subtype is “unclassified colitis,” affecting around 10% to 15% of cases of IBD, in which there is difficulty distinguishing between UC and CD, as it exhibits overlapping clinical and histopathological findings of both CD and UC.^4^ Although this classification has been regarded as fundamental for years, molecular and phenotypic relationships among these diseases suggest that IBD should be regarded as a spectrum.^5^ Moreover, microscopic colitis was recognized as a distinct inflammatory bowel disease.^6^

In 2019, there were approximately 4.9 million cases of IBD worldwide, most of which in China and the United States. Global prevalence, deaths, and disability-adjusted life-years were higher in females than in males, with peak prevalence rate at 50 to 54 years for both sexes.^1^ The global prevalence of IBD has risen over the past decades, with rapidly increasing prevalence rates observed in newly industrialized countries, approaching those seen in Western nations.^1^

The clinical picture of IBD patients is shaped not only by gastrointestinal signs and symptoms, but also by multiple pathological findings at other organs that may coexist and further deteriorate the burden on patients’ health and daily life.^7^ IBD can impact nearly every organ, with the most commonly affected systems being musculoskeletal, ocular, hepatobiliary, cutaneous, renal, pulmonary, cardiovascular, and hematopoietic.^8^ This is summarized under the term “extraintestinal manifestations” (EIMs) and should be differentiated from IBD-associated complications such as anemia, osteoporosis, and micronutrient deficiency, which are secondary due to malabsorption and are not related to a distinct inflammatory process.^9^

According to the European Crohn’s and Colitis Organization Guidelines on Extraintestinal Manifestations in Inflammatory Bowel Disease, EIMs can be grouped into 4 distinct categories: **classical** (inflammatory process occurring at distant sites), **associations** (with other immune mediated disorders), **complications** (eg, treatment induced), and **wider associations**.^10^ Depending on their severity and concurrency, the plethora of associated comorbidities have the potential of significantly complicating the disease course and, ultimately, impairing patients’ QoL.^1^^,^^11^^,^^12^ There have been numerous therapeutic options available such as conventional ones (5-aminosalicylic acid, corticosteroids [systemic or local ones (ie, prednisone or budesonide)], immunomodulators) and biological ones (with main the representative anti-tumor necrosis factor [TNF]), but they often fail to control the disease adequately or tend to lose their efficacy over time, leading to the continuous emerge of alternative treatments. To those belong anti-integrin antibodies (vedolizumab [anti-α4β7 integrin]), anti-interleukin (IL)-12/23 antibodies (ustekinumab, risankizumab [selective IL-23 inhibitor]), and small molecules, namely Janus kinase (JAK) inhibitors (tofacitinib, nonselective; upadacitinib, JAK1 selective), sphingosine 1 phosphate (S1P) receptor modulators, and phosphodiesterase-4 (PDE4) inhibitors.^13–16^ However, these drugs are associated with high financial costs, which, combined with the rising worldwide prevalence of IBD, further emphasizes the multifaceted significance of efficiently improving IBD outcomes while controlling associated comorbidities.

During the disease course, IBD patients often suffer from cutaneous manifestations, most common being pyoderma gangrenosum (PG) and erythema nodosum (EN).^12^ The pathophysiological crosstalk between IBD and cutaneous EIMs remains largely still to be elucidated,^17^ and clinicians are summoned to distinguish manifestations linked to disease relapse from side effects caused by treatment. Within this review, the numerous cutaneous EIMs are presented with special focus on their genetics, pathophysiology, and clinical management with the ultimate aim to summarize available data that will further shed light on their relationship with biologic therapies.

## Cutaneous Manifestations of IBD

Cutaneous manifestations (Table 1) are relatively common among IBD patients, with prevalence ranging from 10% to 30%.^18^ Their onset often coincides with disease flares but not invariably, and their prognosis does not always correlate with the IBD’s clinical course. Traditionally, cutaneous EIMs are classified into 4 distinct categories according to their pathophysiologic mechanisms and relationship with the underlying intestinal disease^19^:

**Table 1.** Summary of cutaneous EIM-IBD relationship.

EIM	Pathogenetic mechanisms	Major genes implicated	Genetic causality	Prognostic value in IBD
**Erythema nodosum**	Type IV hypersensitivity reaction to common antigens of intestinal and cutaneous bacteria	*PTGER4*, *ITGAL*, *IKZF1*, *SOCS5*, *CD207*, *ITGB3*, rs6828740	CD might raise EN risk	Less severe IBD, Colonic illness, follows IBD course
**Pyoderma gangrenosum**	Neutrophil activation, Th1/Th2 imbalance	*IL8RA* (mediator of neutrophil migration), *MUC17*, *MMP24*, *WNK2*, *DOCK9*, *PRDM1*, and *NDIFIP*, as well as SNPs, such as rs6828740	Associated with CD and family history of UC	May or may not follow IBD course
**Sweet syndrome**	Neutrophil activation via TNF-α, IL-8 and photoinduction	Possibly *HLA-B54*	Under investigation	Follows IBD course, colonic involvement, especially in CD
**Hidradenitis suppurativa**	Gut-skin axis, upregulated TNF-α and IL-17, immune dysregulation	*ELOVL7* (protective role), *SULT1B1*, *SULT1E1*	Raised risk in UC and CD via direct causality	Ileocolonic or perianal disease in CD, anti-TNF related, Thiopurine-related
**Psoriasis**	Decreased gut microbiota diversity, altered Th1 and Th17 immune pathways	At least 112 DEGs	Bidirectional	Undetermined
**Rosacea**	Gut-skin axis, immune dysregulation/activation	*IL4*, *IRF1*, and *NEAT1* (under investigation), rs763035	Undetermined, IBD might be causal factor of rosacea	Linked to IBD flares, can be anti-TNF related
**Atopic dermatitis**	Gut microbiota alterations, low SCFA	**CD:** 2q12.1 (*IL1RL1*, *IL18R1*, *IL18RAP*), 11q13.5 (*LRRC32*), 17q21.2 (*STAT3*), 20q13.33 (*RTEL1*, *TNFRSF6B*), **UC:** 4q27, 11q13.5, 20q13.33	Undetermined, possibly bidirectional	Might raise risk of IBD if AD manifests early in life
**Acne conglobata**	Unclear	Potentially *PSTPIP1*	Unknown	Undetermined, evidence of adalimumab-related AC
**CLE**	Unclear	Multiple genetic loci on chromosomes 10, 19, and 22	Higher prevalence in CD, no causality demonstrated	Undetermined, anti-TNF-related
**Vitiligo**	Cell-mediated immunity, endoplasmic reticulum stress	*IL-2RA*, *PTPN22*, *CCR6*, *ZMIZ*	No causality demonstrated	Undetermined
**Chronic urticaria**	↓ Mast cell activation threshold due to gut microbiome alterations	Unknown	No causality demonstrated	Undetermined
**Leukocytoclastic vasculitis**	Unclear	Unknown	Unknown	Mostly independent, **CD:** mostly anti-TNF related
**Alopecia areata**	Unclear, role of gut dysbiosis under investigation	*BTNL2*, *HLA*-*DQA1*, *KLRK1*, *MICA*, *TNFA*, *GARP* (*LRRC32*)	No causality demonstrated	Undetermined
**Skin cancer**	Chronic inflammation, immune dysregulation, oxidative stress	**NMSC:** rs7773324-A (*DUSP22; IRF4*), rs1847472-C (*BACH2*) rs72810983-A (*CPEB4*), rs2476601-G (*PTPN22*) **MSC:** rs61839660-T (*IL2RA*)	IBD causal factor of NMSC	Treatment related (anti-TNF, thiopurines, MTX)

*Abbreviations:* AC, acne conglobata; AD, atopic dermatitis; CD, Crohn’s disease; CLE, cutaneous lupus erythematosus; DEG, differentially expressed gene; EIM, extraintestinal manifestation; EN, erythema nodosum; IL, interleukin; MSC, melanoma skin cancer; MTX, methotrexate; NMSC, nonmelanoma skin cancer; PG, pyoderma gangrenosum; SCFA, short-chain fatty acids; SLE, systemic lupus erythematosus; SNP, single nucleotide polymorphism; SS, Sweet syndrome; Th, T helper; TNF, tumor necrosis factor; UC, ulcerative colitis.

1. **Reactive**: Different histopathologic features but same pathophysiologic mechanisms with underlying disease (EN, PG, Sweet syndrome [SS], oral lesions with distant localized UC/CD).
2. **Specific**: Share similar histopathologic features with the underlying intestinal disease but arise outside of the gastrointestinal tract (extraintestinal CD: oral, perianal, or vulvar CD).
3. **Associated**: Mucocutaneous disorders of inflammatory or autoimmune nature that are not directly linked pathophysiologically to IBD, but they may coexist and share risk factors (hidradenitis suppurativa [HS], psoriasis, atopic dermatitis [AD], acne conglobate [AC], rosacea, vitiligo, alopecia areata [AA], leukocytoclastic vasculitis [LCV], systemic lupus erythematosus [SLE], polyarteritis nodosa, epidermolysis bullosa acquisita).
4. **Complications**: Treatment-induced cutaneous manifestations (psoriasiform lesions, infections, nonmelanoma skin cancer, exfoliative dermatitis, Stevens-Johnson syndrome/toxic epidermal necrolysis, drug reaction with eosinophilia and systemic symptoms).

### Reactive (classical)

#### Erythema nodosum

##### Epidemiology

EN is the most common cutaneous EIM of IBD, with an overall prevalence of 1% to 15% in IBD patients (7%-15% in CD, 2.8%-10% in UC).^20^ However according to a recent meta-analysis, the reported prevalence in CD and UC is 3% and 1%, respectively.^12^ It tends to be more common in women and is also linked to colonic illness and less severe IBD, following typically the clinical course of the latter.^19^

##### Pathophysiology

The known causes of EN are numerous and include infections, drugs, malignancies, various autoimmune/autoinflammatory conditions, and pregnancy.^21^ Pathogenesis is complex, involving a type IV (delayed) hypersensitivity reaction to a variety of antigens. Histopathology reveals lymphocyte, histiocyte, and neutrophil infiltration, with neutrophils predominating in early septal inflammation and lymphocytes along with multinucleated giant cells in late-stage lesions.^21^ Overexpression of T helper 1 (Th1) cytokines (IL-2, interferon γ [IFN-γ]) in EN lesions and serum indicate a polarized Th1 response, whereas high levels of neutrophil-related cytokines (IL-6, IL-8, TNF-α) are also present. However, IL-17 and Th17 cells seem to play a minimal role in primary EN, though they might be upregulated in EN secondary to IBD. Adhesion molecules and immune complexes also contribute to EN development.^22^

##### Clinical presentation

EN is a mostly septal panniculitis with no accompanying vasculitis that presents clinically with symmetric, tender, erythematous, warm nodules and raised plaques (1-5 cm in diameter) that never ulcerate, and heal without atrophy or scarring.^10^^,^^21^ EN lesions are usually located on the knees, shins, and ankles but can also appear on thighs, forearms, and rarely on the neck and face.^10^^,^^21^

##### Diagnosis

Diagnosis is clinical. Biopsy should be considered in persistent cases (>8 weeks) or in atypical/ulcerated lesions.^23^

##### Treatment

Generally, EN lesions self-resolve in 3 to 4 weeks or up to 6 weeks in more severe cases.^24^ Thus, treatment should focus on controlling the underlying disease.^10^^,^^20^ Symptomatic supportive treatment such as bed rest, compression, elevation, analgesics, and potassium iodide should suffice in mild cases.^22^ If lesions are very painful, possible options include short courses of oral corticosteroids, hydroxychloroquine, and anti-TNF agents.^10^^,^^20^^,^^22^ Ustekinumab might also be effective in EN associated with CD.^25^

##### Relevance to IBD

In the context of IBD, the exact pathophysiologic link between the two diseases remains uncertain. Studies indicate that there is a (delayed) type IV hypersensitivity reaction to common antigens of intestinal and cutaneous bacteria, but there is also evidence of genetic associations.^10^ Known IBD susceptibility loci like *PTGER4*, *ITGAL*, *IKZF1*, *SOCS5*, *CD207*, *ITGB3*, and rs6828740 have been found to be associated with EN.^26^ Moreover, a recent study by Zhu et al^27^ demonstrated that (at least in the European population) IBD—and especially CD—raises the risk of EN without reverse causality. Gut microbiota has been hypothesized to play a role in IBD-related EIMs (EN being no exception) also via an impaired gut barrier, microbial translocation, and microbiota-related metabolites,^28^ but to date there is not enough data on these mechanisms for EN or other cutaneous manifestations.^29^ Even though the appearance of EN in IBD patients might be a marker of more systemic disease and lead to faster initiation of therapy, its role as a prognostic factor in IBD treatment outcomes has not been established, yet.

#### Pyoderma gangrenosum

##### Epidemiology

PG is the second most common cutaneous EIM, with reported prevalence ranging from 0.6% to 2.6% among IBD patients (Figure 1). In a systematic review and meta-analysis, PG was found significantly associated with female sex, CD, and EN, as well as with ocular and joint EIMs.^30^ It is also more common among Black Africans and those with a positive family history of UC.^31^

**Figure 1.**
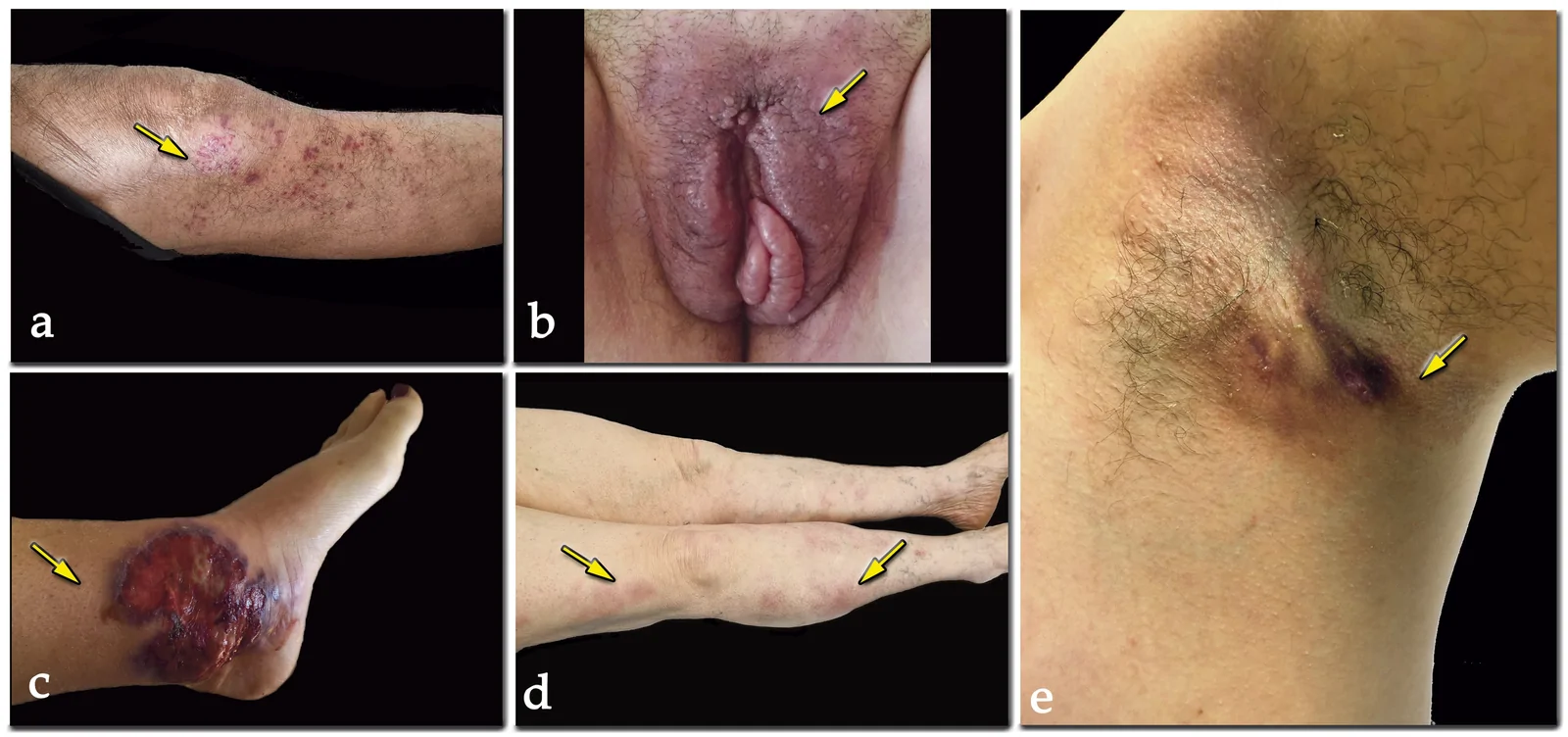
(A) Herpes zoster: erythematovesicular rash with partial crusting on the right lower limb of a 70-year-old male, distributed along the L5 dermatome (rare). (B) **A** 42-year-old woman presented with vulvar Crohn’s disease manifesting as multiple small abscesses and verrucous, wart-like lesions resembling genital condylomata, without evidence of inguinal lymphadenopathy. (C) Pyoderma gangrenosum: a well-demarcated, ulcerated lesion with characteristic violaceous borders on the medial ankle of a 39-year-old woman with Crohn’s disease. (D) Multiple tender bilateral erythematous subcutaneous nodules on the legs of a 50-year-old woman. (E) Hidradenitis suppurativa in the left axilla of a 35-year-old male patient with Crohn’s disease with visible sinus tract openings (ducts) draining purulent material. Arrows indicate characterestic lesions of each patient.

##### Pathophysiology

Pathophysiology of PG remains unclear. Current data indicate that the implicated mechanisms are complex and involve pathogenic genes like *PTPN6* and *PSTPIP1*, chromosomal alterations, immune system dysregulation, autoinflammation, and autoantigens from damaged keratinocytes.^32^ The major mediators of PG are dysregulated neutrophils and T cells (predominantly Th1), with multiple cytokines such as IL-1α, IL-25, IL-17, IL-23, IL-6, IL-8, CCL3, CCL5, and IL-16 triggering inflammatory pathways.^32^

##### Clinical presentation

PG is a noninfectious neutrophilic dermatosis that presents clinically as a papule or pustule that progresses to an ulcer with erythematous to violaceous undermined borders and purulent base.^33^ There is a predilection for the trunk, extensor surfaces, and peristomal areas and the lesions can show pathergy.^10^^,^^33^ There are 6 clinical variants: ulcerative (the most common), pustular, bullous, vegetative, peristomal, and postoperative.^32^^,^^33^ Pustular PG is almost exclusively seen in exacerbation of acute IBD (usually UC) and it is characterized by a generalized, painful eruption of sterile pustules with erythematous halo (centered on hair follicles) on the trunk, face, and limbs.^33^^,^^34^

##### Diagnosis

PG is considered a diagnosis of exclusion because there are no specific laboratory or clinical findings.^34^^,^^35^ Biopsy should be avoided because it may invariably cause an enlargement of the lesion due to pathergy.

##### Treatment

PG can be unpredictable and severely affect QoL via its numerous complications; therefore, early diagnosis and effective treatment in collaboration with dermatologists are crucial.^20^ Management of PG (Table 2) requires wound care (gentle cleansing—no debridement due to pathergy, sterile dressings), topical therapy (superpotent corticosteroids, tacrolimus), intralesional therapy (intralesional corticosteroids, methotrexate [MTX], and ciclosporin A), and systemic therapies (corticosteroids [oral prednisone 0.5-2 mg/kg/day or oral prednisolone 0.75 mg/kg/day], pulse corticosteroids [0.5-1 g methylprednisolone/day for 1-5 days], other immunosuppressants [ciclosporin A, mycophenolate mofetil, MTX, and azathioprine], dapsone, minocycline, intravenous immunoglobulin, anti-TNF [infliximab- the first drug with randomized controlled trial for PG,^36^ and adalimumab], ustekinumab, IL-1 antagonists [canakinumab and anakinra], IL-23 inhibitors [guselkumab, tildrakizumab, risankizumab], and tofacitinib [JAK inhibitor]).^32^^,^^37^^,^^38^ Although IL-17 inhibitors may be efficacious in PG, they should not be used in IBD patients due to reports of IBD exacerbation and new-onset IBD.^39^^,^^40^

**Table 2.** Clinical summary of cutaneous EIMs in IBD.

EIM	Prevalence	Key clinical features	First line treatment	Biopsy indications
**Erythema nodosum**	Most common EIM in IBD (1%-15%), CD>UC	Symmetric, tender, erythematous, warm nodules and raised plaques (1-5 cm in diameter) that never ulcerate. Heal (self-resolving) without atrophy or scarring	Bed rest, compression, elevation, analgesics, potassium iodide in mild cases, oral corticosteroids in very painful lesions	Biopsy rarely needed; consider if atypical presentation (eg, ulceration, nontender lesions) or to rule out other panniculitides
**Pyoderma gangrenosum**	Second most common EIM in IBD (0.6%-2.6%)	Papule or pustule that progresses to an ulcer with erythematous to violaceous undermined borders and purulent base	Wound care: no debridement, systemic corticosteroids	Biopsy not recommended due to pathergy, perform to exclude malignancy in atypical presentation
**Sweet syndrome**	<0.3% of IBD patients	Tender erythematous to violaceous papules, plaques or nodules in a febrile patient with leukocytosis	Control of IBD, topical/intralesional corticosteroids, systemic corticosteroids (oral prednisone 0.5-1 mg/kg/day)	Biopsy recommended in atypical cases (dense neutrophilic infiltrate) with bacterial, fungal, and mycobacterial cultures to rule out infection
**Hidradenitis suppurativa**	Not determined (<5%)	Relapsing painful erythematous papules and nodules, malodorous discharge, abscesses, interconnected sinus tracts, and scars in the axillary, inguinal, and anogenital regions	Analgesics, wound care, smoking cessation and weight loss, systemic antibiotics	Biopsy not recommended
**Psoriasis**	3.6% of CD and 2.8% of UC patients, 43.5% increased risk in UC	Scaly erythematous papules, plaques, and rarely sterile pustules	Topical or systemic treatments depending on clinical variant, avoid IL-17A inhibitors	Biopsy recommended in pustular/erythrodermic forms and in atypical cases/potential overlap with other dermatoses
**Rosacea**	Not determined in IBD, increased risk (especially in UC)	Erythema, telangiectasias, papules, pustules, phymatous changes, flushing	Topical or systemic agents in combination or alone	Consider biopsy in granulomatous rosacea or to rule out lupus
**Atopic dermatitis**	Undetermined in IBD	Eczematous-pruritic patches/plaques	Emollients, topical corticosteroids, topical pimecrolimus	Consider biopsy in treatment-resistant cases
**Acne conglobata**	Rare, increased risk in IBD	Grouped comedones, nodules, cysts, abscesses, draining sinus tracts, scars	Combination therapies-oral isotretinoin (0.5-1.0 mg/kg/day)	Biopsy not recommended
**CLE**	Not determined	Photosensitivity, macules/papules, annular/polycyclic plaques, blisters, exfoliative erythroderma	Diligent photoprotection, topical/systemic agents	Biopsy recommended to confirm diagnosis (interface dermatitis, dermal mucin deposition)
**Vitiligo**	0.3% of IBD patients (UC=CD)	Completely amelanotic areas	Topical potent or highly potent corticosteroids, topical calcineurin inhibitors	Biopsy rarely needed (total loss of epidermal melanocytes)
**Chronic urticaria**	Unknown	Transient, recurrent pruritic wheals of various sizes and shapes, angioedema, or both	Second generation H1-antihistamines, systemic corticosteroids in acute episodes	Biopsy rarely needed, recommended in atypical cases or lesions lasting >24 h to rule out urticarial vasculitis
**Leukocytoclastic vasculitis**	Very rare	Symmetric palpable purpura on the lower extremities	Elevation-compression, systemic corticosteroids	Full-thickness punch biopsy required for diagnosis (lesions <48 h old, but not too early), separate biopsy for direct immunofluorescence.
**Alopecia areata**	Rare (0.6% in IBD patients)	Nonscarring, patchy hair loss	Intralesional corticosteroids, potent topical corticosteroids	Punch biopsy (4 mm) at the margin of a lesion in diagnostic uncertainty
**Skin cancer**	Increased NMSC risk, inconsistent reports regarding MSC	Depends on type of malignancy	Complete resection	Biopsy required to confirm malignancy

Abbreviations: AC, acne conglobata; AD, atopic dermatitis; CD, Crohn’s disease; CLE, cutaneous lupus erythematosus; EIM, extraintestinal manifestation; EN, erythema nodosum; MSC, melanoma skin cancer; NMSC, nonmelanoma skin cancer; PG, pyoderma gangrenosum; LE, lupus erythematosus; SS, Sweet syndrome; UC, ulcerative colitis.

Current treatment modalities (Figure 2) focus on achieving immunosuppression and, in PG associated with underlying disease, control of the latter is very important.^32^ Promising drugs in this direction are novel JAK inhibitors such as tofacitinib (approved for UC), baricitinib, upadacitinib, and ruxolitinib,^32^ as well as IL-36 inhibitor spesolimab, which is under investigation for IBD.^41^^,^^42^

**Figure 2.**
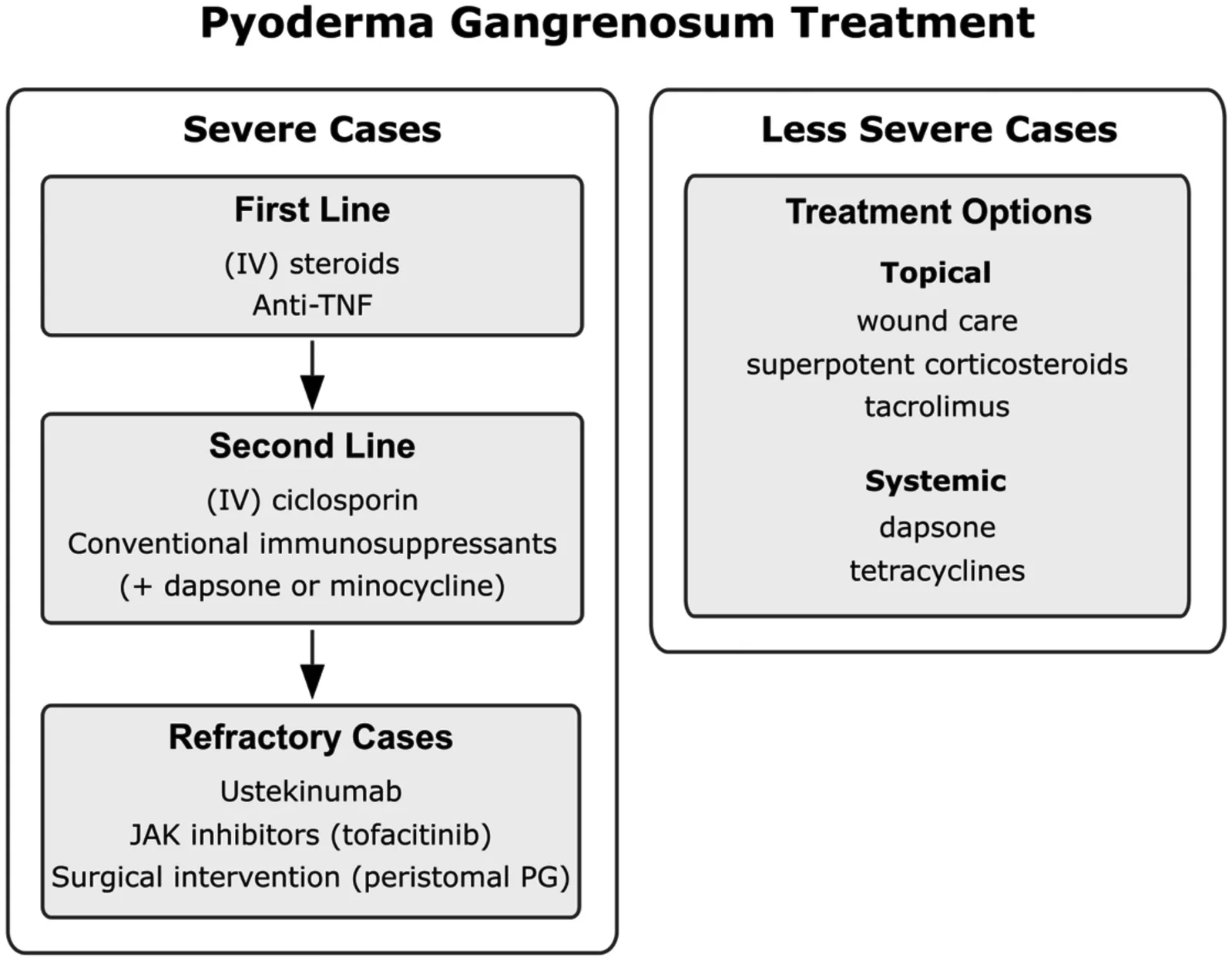
Pyoderma gangrenosum (PG) treatment according to 2024 European Crohn’s and Colitis Organization Guidelines on Extraintestinal Manifestations.^10^ IV, intravenous; TNF, tumor necrosis factor.

##### Relevance to IBD

The exact genetic link between PG and IBD is still elusive, but several known IBD susceptibility loci have been associated with PG, including *IL8RA* (mediator of neutrophil migration), *MUC17*, *MMP24*, *WNK2*, *DOCK9*, *PRDM1*, and *NDIFIP*, as well as single nucleotide polymorphisms, such as rs6828740.^26^ There is also evidence that family history of UC is independently associated with PG.^31^

The role of PG as a prognostic factor in IBD is still under investigation, but its appearance can lead to early diagnosis of IBD and therefore, better outcomes.^43^ Its clinical course may or may not follow the intestinal disease, sometimes being independent and sometimes even preceding IBD onset in approximately 15% of patients.^10^ Colonic involvement of underlying disease and increased risk of developing another EIM (mainly inflammatory ocular manifestations and arthritis) have been reported in IBD patients.^26^^,^^44^ A retrospective cohort study by Panganiban et al^45^ exhibited that PG is associated with increased risk of pouch failure in UC patients with ileal pouch-anal anastomosis.

#### Sweet syndrome

##### Epidemiology

SS is a relatively rare cutaneous EIM of IBD (<0.3% of IBD patients) without specific predilection to UC or CD, that can onset before the diagnosis of IBD (5.3% of cases), alongside IBD diagnosis (29.5%) or after diagnosis (64.2%).^46^ It is also more frequent in association with colonic involvement, especially in CD patients.^46^ It is more common in 30- to 60-year-old female patients but can affect both sex at any age.^47^

##### Pathophysiology

According to the etiology, SS is usually classified as (1) classic (idiopathic), (2) malignancy associated, and (3) drug induced.^47^ Recognized causes are infections (viral, bacterial, fungal), vaccinations (including COVID-19 vaccines), immunodeficiency, inflammatory disorders (IBD, systemic lupus erythematosus, etc.), pregnancy, malignancies, and drugs. SS as an EIM of IBD is classified as the classic type.^47^ Nevertheless, there is a relatively frequent drug-induced form of SS due to azathioprine (AZA) use in the treatment of IBD—which should be distinguished from IBD-related SS—because they differ in epidemiology, presentation, and prognosis; the AZA-related form is considered a hypersensitivity reaction (exact antigen unknown) independent of thiopurine S-methyltransferase levels, develops within a month after AZA initiation, and responds well to AZA cessation, with lower recurrence risk compared with other SS forms.^46^^,^^48^

##### Clinical presentation

SS is a neutrophilic dermatosis characterized by rapid development of tender erythematous to violaceous papules, plaques, or nodules in a febrile patient with leukocytosis.^47^^,^^49^ In recent years, clinical presentation has been regarded as a spectrum; bullous SS, cellulitis-like SS, necrotizing SS, and neutrophilic dermatosis of the dorsal hands are recognized clinical variants.^50^ In general, lesions tend to appear on the upper extremities, face, and neck and less frequently on the trunk and lower extremities.^51^ Interestingly, SS’s incidence seems to be increasing in pediatric populations in which the lesions follow a different distribution favoring the lower and upper extremities, while atypical variants prevail.^50^

##### Diagnosis

SS is a diagnosis of exclusion; therefore, biopsy and cultures of the lesions are recommended.^47^ Histopathologically, SS is characterized by dense diffuse neutrophilic infiltrate of the upper dermis, variably admixed with eosinophils, histiocytes, and lymphocytes, with or without dermal papillary edema.^46^^,^^51^ If LCV is present, it most probably is secondary.^51^ Elevated erythrocyte sedimentation rate, C-reactive protein (CRP), and neutrophilia are the most common blood abnormalities, while anemia-associated markers and thrombocytosis are not as frequently observed.^46^

##### Treatment

Because IBD-related SS follows the course of the underlying disease, treatment should focus on controlling the latter. Ιn the majority of cases, the first therapeutic choice are systemic corticosteroids (eg, prednisone 0.5-1 mg/kg/day) due to the satisfactory response rates that they exhibit.^10^^,^^46^^,^^50^ In milder cases, topical or intralesional corticosteroids may be used.^51^ Biologics (including infliximab, golimumab, ustekinumab, and vedolizumab) are the second most common option and may be used alongside corticosteroids.^10^ AZA, granulocyte-monocyte apheresis, nonsteroidal anti-inflammatory drugs (NSAIDs), dapsone, colchicine, ciclosporin, and MTX, as well as indomethacin, acitretin, and thalidomide, are alternatives reported in the literature.^10^^,^^50^

##### Relevance to IBD

Regarding IBD-associated SS, the common pathophysiologic mechanisms and genetic predispositions are still under investigation. *HLA-B54* has been linked with SS,^47^ and although there is no strong evidence connecting it directly with IBD, there are reports linking the aforementioned polymorphism to autoimmune hepatitis,^52^^,^^53^ which can also emerge as EIMs of IBD.^54^ Photoinduction with proinflammatory cytokines such as TNF-α and IL-8 leading to activation of neutrophils in the skin has been proposed as a potential pathogenetic mechanism.^46^

Clinically, SS tends to occur early in IBD course and parallels the underlying disease activity; control of the latter seems to lead to fewer recurrences of SS.^46^ Moreover, SS could be a positive prognostic factor for the occurrence of bowel penetrations or strictures in CD patients with colonic involvement.^46^

### Associations

#### Hidradenitis suppurativa

##### Epidemiology

The prevalence of HS in general population is not determined, but estimates range from 0.00033% to 4.1%.^55^ Similarly, there is not enough data on prevalence in IBD patients, possibly due to underreporting and delayed diagnosis.^56^ A 2019 systematic review and meta-analysis by Chen et al^57^ reported an elevated risk of HS in CD patients (pooled odds ratio [OR], 2.12; 95% confidence interval [CI], 1.46-3.08) and in UC patients (pooled OR, 1.51; 95% CI, 1.25-1.82) when compared with non-IBD control subjects.

##### Pathophysiology

The pathophysiology of HS is not fully understood; current evidence supports that the first event in pathogenesis is follicular hyperkeratosis, which leads to occlusion, rupture, and subsequent inflammation of nearby tissues due to release of follicular contents.^58^ Bacterial dysbiosis and leucocytic infiltration may also play a role in abscess formation.^59^ Studies report microbiome alterations in lesional and unaffected skin of HS patients, likely contributing significantly to pathogenesis.^55^ In short, release of TNF, IL-1β, IL-17A, and IL-17F from inflammatory dendritic cells, macrophages, and T cells leads to production of chemokines CXCL1, CXCL8, and CCL20 by keratinocytes, promoting further immune cell infiltration into the area and chronic inflammation, notably through continuous neutrophil recruitment via lipocalin-2.^55^^,^^59^

Acknowledged lifestyle predisposing factors include smoking, psychological stress, and obesity.^58^ Positive family history is common among HS patients, and there is evidence of inheritance of specific mutations in the chromosome 1p21.1-1q25.3 region, as well as nicastrin (*NCSTN*), presenilin 1 (*PSEN1*), and presenilin enhancer (*PSENEN*) genes.^58^ Interestingly, in a case-control study by Neri et al,^60^ obesity, smoking, and family history of HS were less frequent in IBD patients with HS.

##### Clinical presentation

HS (Figure 1) is defined by the Hidradenitis Suppurativa Foundation as “chronic, inflammatory, recurrent, debilitating, skin follicular disease that usually presents after puberty with painful deep-seated, inflamed lesions in the apocrine gland-bearing areas of the body, most commonly, the axillary, inguinal, and anogenital regions.”^61^ These lesions may be malodorous due to discharge, and they progress to abscesses, interconnected sinus tracts, and scars, severely affecting QoL.^58^^,^^62^ Prodromal erythema, paresthesia, and pruritus may also manifest before flares.^63^ There are 5 recognized clinical phenotypes: regular HS (most common), frictional furuncle (sites with increased friction in obese patients), scarring folliculitis, HS conglobata, and syndromic HS.^62^

##### Diagnosis

Diagnosis is clinical, as there are no available pathognomonic tests.^55^ Noninvasive biomarkers have been found (amyloid A, CRP, IL-2R, lipocalin-2, IL-17A, IL-17F, human beta-defensin, and fecal calprotectin), but none of them is recommended for clinical use.^59^

Because of the phenotypic variations of HS, multiple diagnostic techniques have been designed, including Hurley staging, Hidradenitis Suppurativa Clinical Response, Modified Sartorius Score, Physician Global Assessment Tool for HS, Hidradenitis Suppurativa Severity Index, International Hidradenitis Suppurativa Severity, IHS4-55, and Acne Inversa Severity Index.^58^^,^^64^ Traditional ultrasound, color Doppler and magnetic resonance imaging are valuable tools that can aid in detection and classification of lesions that have not yet fully manifested, greatly improving clinical decisions.^65–67^

##### Treatment

Treatment consists of analgesic-alleviating measures (paracetamol, NSAIDs, drainage of lesions, pregabalin, gabapentin, venlafaxine, duloxetine, antidepressants, opioids), wound care (warm compresses followed by bleach baths and Epsom salt baths), topical therapies (resorcinol 15%, clindamycin 1%, keratolytics, and skin cleansers), psychological support, lifestyle modifications (smoking cessation, weight loss), and in severe cases, systemic therapies (antibiotics, hormonal therapy, isotretinoin, ciclosporin, MTX, oral steroids [high dose for flare control, low dose over time to avoid adverse events (AEs)], and biologics [adalimumab, secukinumab, and potentially infliximab, etanercept, anakinra, canakinumab, bimekizumab, guselkumab, risankizumab, rituximab, ustekinumab, apremilast, and povorcitinib]) and surgery (local excision, deroofing, extensive excision, lasers, primary suturing, skin grafts, secondary healing, platelet-enriched plasma, and vacuum-assisted closure).^58^ Several new classes of drugs are currently undergoing clinical trials, offering the promise of improved outcomes for patients with HS.^68^^,^^69^

In HS associated with IBD, systemic treatment with anti-TNF, specifically adalimumab (first line) and infliximab, can effectively manage both conditions. Ustekinumab trials are insufficient to fully support its adoption into clinical practice.^10^ AZA may also be an effective option for HS and its role is already established in IBD.^70^ Among small molecule inhibitors, tofacitinib and sirolimus also seem promising.^71^ Systemic retinoids (especially acitretin) are effective for HS lesions^72^ and are low cost, making them a viable option for eligible patients (category X teratogens). However, they do not treat IBD and are not first-line therapy according to the S2k guideline for the treatment of HS.^73^ On the other hand, biologics and newer drugs lack sufficient evidence to be regarded as effective options for HS,^59^ and treating physicians should carefully consider biologics’ potential for serious AEs.^74^

##### Relevance to IBD

The mechanisms linking IBD and HS are not yet fully defined, but there is significant association of HS with IBD and its subtypes.^75^ IBD is a causal factor for HS, but the reverse is not true according to a 2-sample bidirectional Mendelian randomization study by Bao et al.^76^ Furthermore, HS onset has been reported in IBD patients after treatment with TNF antagonists (infliximab, adalimumab, golimumab) and after thiopurines (AZA, 5-mercaptopurine).^60^ Alterations of microbiota (gut-skin axis), upregulated TNF-α and IL-17 levels, and immune dysregulation are shared pathogenetic factors in both diseases,^60^ whereas common associated genes include *ELOVL7* (protective role), *SULT1B1*, and *SULT1E1.*^77^

Specifically in CD patients, HS is linked with ileocolonic or perianal disease.^78^ Contiguous perianal CD should be differentiated from HS, as both can present similarly, posing a diagnostic challenge.^79^ No increased risk in UC has been reported.

#### Psoriasis

##### Epidemiology

Psoriasis affects adults more than children, with global prevalence ranging from 0.14% to 1.99% and higher rates are seen in wealthier populations, though underreporting remains an issue.^80^ Psoriasis occurs in 3.6% of CD and 2.8% of UC patients, while 0.7% of psoriatic patients experience CD and 0.5% UC.^81^ Bidirectional causality with IBD has been demonstrated,^82^ with a 43.5% increased risk of psoriasis in UC patients (OR, 1.435; 95% CI, 1.274-1.831; *P < .*001).^83^ Psoriasis or psoriasiform lesions may also develop as a side effect of biologics, primarily with anti-TNF (see Side Effects of IBD Therapies for details).^84^

##### Pathophysiology

There is a growing body of evidence supporting shared pathogenesis and genetic basis for IBD and psoriasis including at least 112 identified overlapping differentially expressed genes,^82^^,^^85^ the JAK/STAT pathway, macrophages M0 and M1, CD4+ T cells, and FOS (biomarker).^86^

##### Clinical presentation

Psoriasis is a chronic inflammatory papulosquamous disease, characterized by scaly erythematous papules, plaques, and rarely sterile pustules.^87^ There are 6 clinical variants: vulgaris (plaque psoriasis; most common), inverse, guttate, palmoplantar pustulosis, erythrodermic, and generalized pustular.^87^^,^^88^ Depending on the severity, it can seriously affect QoL, both mentally and physically, with its numerous comorbidities (cardiovascular, musculoskeletal, etc.).^89^

##### Diagnosis

Diagnosis is mostly clinical. Biopsy may be needed for confirmation in pustular psoriasis, erythrodermic psoriasis or atypical cases where other diseases should be ruled out (eg, mycosis fungoides).

##### Treatment

Treatment of psoriasis is based on the specific clinical variant, severity, and comorbidities and consists of topical/oral systemic therapies, phototherapy, and biologics (anti-TNF, anti-IL-17, anti-IL-23, and anti-IL-12/23 common p40 unit, ustekinumab).^87^ Anti-TNF agents are recommended due to their established safety and efficacy profiles across both diseases, even though IL-17 and IL-23 inhibitors show greater efficacy.^90^ On the other hand, avoidance of IL-17A inhibitors is advisable due to possible exacerbations of intestinal disease.^91^

##### Relevance to IBD

A recent study by Guo et al^92^ supported the “gut-skin axis” concept by demonstrating that IBD causally increases the risk of psoriasis without observation of the reverse; thus, intestinal inflammation might precede and trigger skin inflammation.

#### Rosacea

##### Epidemiology

Epidemiology is unclear. The estimated global prevalence in the general adult population is approximately 5.5%,^93^ while IBD (especially UC) patients are reportedly 86% more likely to develop rosacea.^94^ During intestinal flares and after initial diagnosis, risk for rosacea may be up to trifold.^95^ Although studies suggest a bidirectional association of IBD and rosacea,^94^ Mendelian randomization shows that IBD is the causal factor, not the reverse.^96^^,^^97^

##### Pathophysiology

IBD and rosacea share genetics and underlying pathophysiologic mechanisms.^94^ IBD genes possibly contributing to rosacea are *IL4*, *IRF1*, and *NEAT1.*^96^ A polygenic single nucleotide polymorphism named rs763035 has also been linked to both conditions.^97^ Gut-skin axis and immune dysregulation/activation (Th1, Th17, B cells and macrophages, IL-1b, TNFα, IFNγ, and IL-17) are commonly implicated.^98^

##### Clinical presentation

Rosacea is a chronic inflammatory disease of the central face, characterized by erythema, papules, pustules, telangiectasias, phymatous changes, and flushing,^99^^,^^100^ with substantial effect on QoL.^101^

##### Diagnosis

Diagnosis is clinical and correct classification of the disease, albeit difficult in skin of color due to erythema concealment by melanin, is critical for efficient management (Table 2) and personalized treatment. The ROSCO (Rosacea Consensus) 2019 consensus suggests the phenotype approach in rosacea.^100^

##### Treatment

Treatment consists of topical and systemic agents in combination or alone.^99^^,^^102^^,^^103^ Rifaximin, an oral antibiotic with virtually no systemic absorption,^104^ has revealed promising results in treating rosacea patients specifically with small intestinal bacterial overgrowth.^105^^,^^106^ Small intestinal bacterial overgrowth is common in IBD patients and treating the former has been efficacious in reducing gastrointestinal symptoms of UC and CD.^107^

##### Relevance to IBD

Evidence on the potential causal relationship between IBD and rosacea is contradictory,^96^ although they share pathogenetic mechanisms (see Pathophysiology). Notably, rosacea can manifest as an AE of anti-TNF treatment.^108^

#### Atopic dermatitis

##### Epidemiology

AD affects 2.6% of global population (2.0% of adults and 4.0% of children), with higher risk associated with female sex.^109^

##### Pathophysiology

The pathophysiology of AD is complex. In short, dysregulation of both the innate and adaptive immune systems triggers the skin barrier impairment, which is an indirect result of immune responses to irritants and allergens. This mechanism involves pattern recognition receptors (PRRs) such as Toll-like receptors, the IL-23/IL-17 axis along with Th2 polarization,^110^ and Langerhans cells.^111^ Genetics (ie, FLG gene mutations), diet, and environmental factors are acknowledged contributors to AD, some of which are also implicated in IBD.^110^ Four CD risk loci (2q12.1 [*IL1RL1*/*IL18R1*/*IL18RAP*], 11q13.5 [*LRRC32*], 17q21.2 [*STAT3*], 20q13.33 [*RTEL1*/*TNFRSF6B*]) and 3 UC loci (4q27, 11q13.5, 20q13.33) have been associated with AD.^112^ Short-chain fatty acids, which are produced by intestinal microbiota and are linked with normal epithelial barrier function, have been found in reduced quantities in inflamed gut mucosa (eg, in IBD),^113^ and low short-chain fatty acids have been associated with AD onset.^114^

##### Clinical presentation

AD is a chronic inflammatory skin disorder characterized by eczematous-pruritic patches/plaques and recurrent skin infections due to defects of skin-barrier function and skin dysbiosis.^110^^,^^115^^,^^116^ The mental/psychological and somatic burden on patients is substantial, due to sleep loss (pruritus) and/or disfiguring lesions.^117^

##### Diagnosis

Diagnosis is mostly clinical.^110^ Skin cultures and biopsies may be needed in atypical/complicated or treatment-resistant cases.

##### Treatment

Treatment of AD includes multiple modalities.^115^^,^^118^ Biologics (dupilumab, tralokinumab, lebrikizumab, nemolizumab) and JAK inhibitors (abrocitinib, upadacitinib, baricitinib) are generally considered better choices than other systemic treatments in terms of efficacy and safety profile, despite the Food and Drug Administration black box warning for all JAK inhibitors.^119^ Although (to our accessible literature) no studies have been conducted on humans, there is evidence of curcumin treating AD in mouse models, via its anti-inflammatory and antioxidant properties.^120^

##### Relevance to IBD

Although evidence suggests a bidirectional relationship between IBD and AD,^121^ studies are contradictory.^122^^,^^123^ Moreover, children with AD appear to have an increased risk of developing IBD later in life.^124^ Consecutively, more studies are needed to fully elucidate this relationship.

#### Acne conglobata

##### Epidemiology

Epidemiology is unknown in IBD.

##### Clinical presentation

AC is considered a severe form of inflammatory acne, characterized by comedones (often multiple), interconnected draining sinus tracts/nodules/cysts, and scarring.^125^ Common locations are the face, arms, upper torso, buttocks, and thighs.^125^

##### Diagnosis

Diagnosis is clinical and biopsy is generally not recommended.

##### Treatment

Because AC is often treatment resistant, combination therapies are usually administered. These include oral isotretinoin (first line, 0.5-1.0 mg/kg/day), oral corticosteroids (prednisolone), oral antibiotics (usually unsuccessful), dapsone (50-150 mg/day), adalimumab, photodynamic therapy, ablative laser, and surgical interventions.^126–131^

##### Relevance to IBD

Prevalence in IBD patients is unknown, but there is evidence of increased risk of AC in this group, possibly due to TNF-α involvement.^132^ However, there is a case report of AC after adalimumab treatment.^133^ Generally, AC is associated with positive family history of severe acne,^134^ which suggests that genetic factors play a role in its pathogenesis. Mutations in *PSTPIP1* gene (also in PG) have been reported in PAPA syndrome, as well as in PAC syndrome,^135^ potentially linking severe acne with IBD. To date, there are no concrete statistical data on common genetic loci/mutations in AC and IBD.

#### Systemic lupus erythematosus

##### Epidemiology

The epidemiology is unknown in IBD.

##### Pathophysiology

SLE is a multisystem autoimmune disease that can affect any organ and is characterized by irreversible tissue damage via production of autoantibodies, complement activation, and cytokine dysregulation.^136^^,^^137^

SLE tends to coexist with other autoimmune diseases such as vasculitides, antiphospholipid syndrome, and IBD.^138^ Its association with IBD is not fully understood, but there is evidence of multiple genetic associations.^139^

##### Clinical presentation

Cutaneous manifestations of SLE vary in morphology, distribution, and histologic findings and are classified as (1) acute (acute cutaneous lupus erythematosus [CLE], 30%-50% of SLE patients), (2) subacute (subacute cutaneous lupus erythematosus [SCLE], 10%-15%), and (3) chronic CLE. Acute CLE is associated with SLE flares and may present as photosensitive malar rash, photosensitive generalized maculopapular rash, or widespread toxic epidermal necrolysis-like blistering.^140^ SCLE presents as annular to polycyclic plaques, papulosquamous rash, or generalized exfoliative erythroderma.^140^

##### Diagnosis

Diagnosis is based on clinical presentation, laboratory findings, and biopsies. The role of rheumatologists is crucial because SLE can mimic a plethora of other diseases with potentially debilitating chronic and irreversible complications.^140^

##### Treatment

Due to the photosensitivity of cutaneous lupus lesions, treatment should begin with diligent photoprotection. Pharmacological treatment consists of topical/systemic agents, depending on the severity of lesions.^141^ Biologics in SLE is a topic of current research, but their efficacy in treating cutaneous lesions is still debated. Among the new targeted therapies, belimumab, rituximab, ustekinumab, and baricitinib appear to be the most promising in this direction.^142^

##### Relevance to IBD

Reported prevalence of CD is higher in SLE patients.^143^ However, a 2-sample mendelian randomization study by Xie et al^144^ concluded that SLE is associated with lower IBD risk in European populations, but no causal relationship could be found in East Asian populations.

#### Vitiligo

##### Epidemiology

Vitiligo’s prevalence in Europe, Japan, and the United States is 1.3%, whereas in Africa, the Middle East, and Latin America it ranges from 0.18% to 5.3% with large geographic variation.^145^^,^^146^ Approximately 0.3% of IBD patients experience vitiligo, with equal distribution between UC and CD.^147^

##### Pathophysiology

Pathogenesis of vitiligo is complex, with cell-mediated immunity and oxidative stress being the core mechanisms leading to apoptosis of melanocytes.^148^ The latest research has revealed the major role of endoplasmic reticulum stress, which is expressed as accumulation of unfolded or misfolded proteins and endoplasmic reticulum structural changes.^149^ At the molecular level, sirtuin 7 was recently found to be highly expressed in vitiligo skin lesions, desuccinylating G6PD and potentially promoting vitiligo progression.^150^ Genetics also play a role in vitiligo pathogenesis; *IL-2RA*, *PTPN22*, *CCR6*, and *ZMIZ* comprise the common genetic background with IBD.^151^

##### Clinical presentation

Vitiligo is a chronic autoimmune disorder characterized by apoptosis of melanocytes, resulting in completely amelanotic skin areas.^152^ The psychosocial burden of the disease is substantial, especially in younger patients and those with darker skin; thus, vitiligo should not be regarded solely as a harmless aesthetic nuance.^153^

##### Diagnosis

Diagnosis is mostly clinical using a dermatoscope (perifollicular pigmentation/depigmentation) and a Wood’s lamp (vitiligo lesions glow blue-white with sharp borders).^152^ Histopathologically, lesions are characterized by total loss of epidermal melanocytes.^152^

##### Treatment

Treatment focuses on disease stabilization/repigmentation and consists of topical and systemic agents, phototherapy, surgery, and other depigmentation treatments.^154^ Notably, biologics such as anti-TNF and anti-IL-17 are not recommended for treatment of vitiligo.^154^

##### Relevance to IBD

No direct causal relationship has been demonstrated between the two diseases.

#### Chronic urticaria

##### Epidemiology

Prevalence of chronic urticaria (CU) in the general population is <1.5%,^155^ but there are no epidemiological data regarding IBD patients.

##### Pathophysiology

Pathophysiology involves mast cell activation and release of histamine and other mediators that lead to vasodilation and edema in dermis (wheals) and/or subcutis (angioedema).^156^ There is increased evidence that gut microbiome alterations influence CU by lowering mast-cell activation threshold, reducing exposure to inhibitory signals, and increasing inflammatory diathesis via metabolites.^157^

##### Clinical presentation

Urticaria is a common cutaneous disease characterized by transient, recurrent pruritic wheals of various sizes and shapes, angioedema, or both.^156^

##### Diagnosis

Diagnosis is based on clinical examination and history taking and, in difficult cases, provocation tests may be utilized. All patients with chronic spontaneous urticaria should have differential blood count, CRP, and/or erythrocyte sedimentation rate. IgG-anti-TPO, total IgE, and more advanced tools like basophil activation testing can help in the diagnostic workup.^156^

##### Treatment

Treatment is focused on recurrence prevention. Avoiding unspecific triggers like stress and NSAIDs could help reduce the frequency of episodes. Symptomatic treatment is based on continuous use of second generation H1-antihistamines (up to 4-fold dose—off label). If antihistamines fail to control recurrences, omalizumab and ciclosporin are second- and third-line options, respectively. Systemic corticosteroids can be used to control acute urticaria, but long-term use is strongly discouraged.^156^ Infliximab and adalimumab have also been used successfully in a CD patient with CU/angioedema.^158^

##### Relevance to IBD

A significant association of CU with various autoimmune diseases was found, including IBD.^159^^,^^160^ However, a bidirectional 2-sample mendelian randomization study by Yang et al^161^ did not find any significant genetic causal relationship between urticaria and IBD.

#### Leukocytoclastic vasculitis

##### Epidemiology

Global prevalence of LCV is unclear; annual incidence was 45 per 1 000 000 in a population-based study, favoring adults in 1:1 male-to-female ratio.^162^ Although prevalence in IBD is unknown, LCV is one of the rarest EIMs.^163^

##### Pathophysiology

Pathophysiology of LCV is characterized by immune complex/cryoglobulin deposition in dermal venules and capillaries, neutrophil recruitment, and subsequent inflammation of small blood vessels, leading to vessel wall damage.^164^

##### Clinical presentation

LCV is a small vessel vasculitis primarily affecting the skin.^164^ Clinically, it presents as symmetric palpable purpura (nonblanching, purple papules, plaques, and rarely, bullae/ulcerating necrotic lesions) predominantly on the lower extremities.^164–166^

##### Diagnosis

Diagnosis is mostly clinical, augmented by laboratory tests, but biopsy is mandatory for confirmation.^164^ The lesions biopsied should be <48 hours old but not too early in order not to miss characteristic LCV findings. Moreover, full-thickness biopsies are essential, and a separate biopsy for direct immunofluorescence is advised.^164^

##### Treatment

Treatment is either symptomatic (elevation and compression stockings) or systemic with corticosteroids (usually rapid response), colchicine, dapsone, AZA, MTX, mycophenolate mofetil, or rituximab, depending on clinical severity.^164^ In case of anti-TNF drug–induced LCV, cessation of the offending drug along with a short course of oral steroids leads to complete cutaneous remission in 4 to 12 weeks, and switching to a different biologic maintains IBD control without vasculitis recurrence.^167^

##### Relevance to IBD

LCV can be associated with a variety of underlying conditions, including infections, medications, malignancies, and autoimmune diseases such as IBD, particularly UC.^164^^,^^165^ In UC patients, LCV is almost always a true association, can precede UC diagnosis, and tends to occur during an intestinal flare, but not exclusively.^165^ In contrast, LCV in CD patients is mostly secondary to biologic therapy, particularly to infliximab and adalimumab.^165^

#### Alopecia areata

##### Epidemiology

AA affects 6 in 1000 IBD patients.^168^ Although rare,^169^ skin of color and Asians seem to be disproportionally affected.^169^

##### Pathophysiology

The exact etiology of AA remains unclear, but it may result from a combination of genetic predisposition and environmental triggers.^170^ Research has suggested a potential link between AA and IBD due to shared immunological pathways with IFN-γ and TNF-α implicated in pathogenesis of both pathologies,^168^ as well as due to multiple shared genotypes.^171^ Among those, *BTNL2*, *HLA-DQA1*, *KLRK1*, *MICA*, *TNFA*, and *GARP* (*LRRC32*) are shared with IBD.^171^

##### Clinical presentation

AA is an autoimmune disease characterized by sudden, nonscarring, patchy hair loss caused by the immune system mistakenly attacking hair follicles.^170^ Rarely, in severe forms or in children, the proximal nail matrix may be affected, causing pitted, longitudinally ridged, and brittle or otherwise deformed nails.^172^

##### Diagnosis

Diagnosis is mostly clinical as the presentation is characteristic with asymptomatic well-circumscribed hair loss. In dermoscopy, “exclamation mark” hairs, vellus hairs, yellow dots, black dots or a combination of them may be appreciated, marking different stages of hair follicle disease.^173^

##### Treatment

Treatment consists of topical and systemic drugs (systemic corticosteroids, MTX, ciclosporin, JAK inhibitors, and mesenchymal stem cell therapy).^170^ Biologics and PDE4 inhibitors are not recommended due to limited efficacy.^174^ JAK inhibitors are the most promising; tofacitinib has proven effective in severe cases with nail involvement, but newer drugs (baricitinib, ritlecitinib) lack robust evidence regarding their effectiveness on nails.^172^ Moreover, although upadacitinib is not approved for AA, it has been used successfully to treat AA concomitant with CD.^175^

##### Relevance to IBD

Despite the aforementioned associations with IBD, no causative relationship has been demonstrated. There is evidence that gut microbiota play an important role, but further studies are needed to establish their significance.^176^

### Complications: Side effects of IBD therapies

Biologics/targeted systemic therapies have revolutionized the treatment of various immune-mediated inflammatory diseases, including IBD, due to their high efficacy and good safety profile. However, their introduction was accompanied with new “paradoxical” AEs, which resemble pathological conditions normally treated by the causative drug.^37^ Paradoxical manifestations (Figure 3) can be systemic or localized.^177^

**Figure 3.**
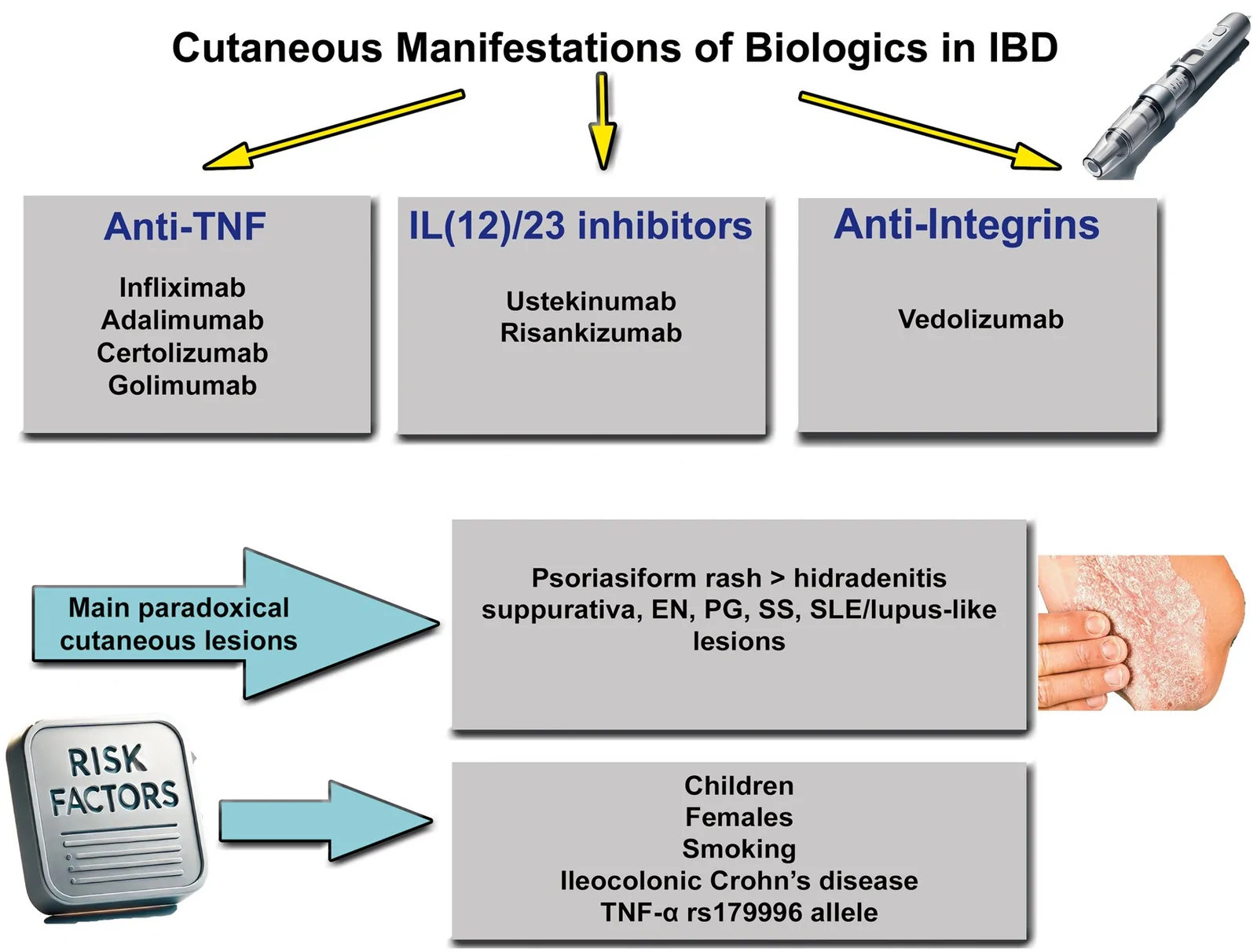
Paradoxical skin manifestations associated with biological therapies in inflammatory bowel disease (IBD) patients. The most common manifestations include anti-tumor necrosis factor (TNF) agents (eg, infliximab, adalimumab), interleukin (IL)-12/23 inhibitors (eg, ustekinumab, risankizumab), and anti-integrins (eg, vedolizumab). Common paradoxical lesions include psoriasiform rash, erythema nodosum (EN), pyoderma gangrenosum (PG), Sweet syndrome (SS), and systemic lupus erythematosus (SLE)–like, hidradenitis suppurativa (HS)–like, and vasculitis-like lesions. Identified risk factors include younger age, female sex, smoking, ileocolonic Crohn’s disease, and the TNF-α rs179996 allele.

Most reports on paradoxical cutaneous manifestations in IBD patients are due to anti-TNF treatment, with 25.9% of patients in prospective studies and 15.7% in retrospective studies presenting with any paradoxical skin lesion, without statistically significant sex predilection.^178^ Pediatric patients seem to be more affected compared with adults, with higher risk linked to younger age of drug initiation.^178^ The documented cutaneous anti-TNF induced paradoxical lesions are psoriasis/psoriasiform rash (most reported), HS, PG, EN, interstitial granulomatous dermatitis, vasculitis, and SLE/lupus-like lesions. Other cutaneous AEs include skin infections, xerosis, eczema, blepharitis, infusion/injection reactions, acne, rosacea, alopecia, and malignancies.^10^^,^^108^^,^^177–180^

Infliximab (67.5%) and adalimumab (30.5%) are the most implicated in development of paradoxical lesions, with much fewer reports on certolizumab (1.7%) and golimumab (0.3%).^178^ Paradoxical psoriasis or psoriasiform lesions are well-known AEs associated with anti-TNF treatment. In a systematic review with meta-analysis by Xie et al,^84^ female sex, younger age, smoking, and ileocolonic CD carried the highest risk for paradoxical psoriasis. Cutaneous lupus–like lesions (in the form of SCLE and chronic CLE) have been reported as AEs of etanercept, infliximab, adalimumab, and certolizumab pegol.^181^

However, anti-TNF drugs are not the only contributors to paradoxical manifestations. There are case reports of vedolizumab-induced PG, SS, and psoriasiform dermatitis in IBD patients.^182–184^ Ustekinumab has rarely been reported to cause paradoxical psoriasis and risankizumab may be helpful in these patients, although there is also a case report of risankizumab-induced psoriasis.^185^ To our knowledge, there is no evidence on whether IL-12/23 inhibitors, JAK inhibitors, S1P receptor modulators, and PDE4 inhibitors can directly induce or treat paradoxical cutaneous manifestations in IBD patients. Baricitinib may be an exception, as there is a case report of baricitinib-induced paradoxical psoriasis, albeit in a rheumatoid arthritis patient.^186^ However, JAK inhibitors have been associated with increased risk for herpes zoster (HZ) (see Herpes Zoster section) and acneiform reactions (9.7% of patients on upadacitinib in a multicenter study, followed by reactivation of cytomegalovirus, thrombosis, herpes simplex, and paraesthesia).^187^

The underlying mechanisms of paradoxical reactions are not fully elucidated, but in essence they are immunological intolerance reactions.^188^ Specifically in the case of anti-TNF cutaneous reactions, cytokine imbalance (increased IFN-α) leads to activation of dendritic cells and subsequent Th17-induced inflammation with increased IL-23, IL-17, and IL-22.^188^ Alterations of gut and skin microbiota may also be implicated in pathogenesis of cutaneous manifestations during anti-TNF treatment, via metabolites or induction of immune response to microbial antigens secondary to impaired gut barrier.^29^^,^^189^ Risk factors associated with psoriasiform lesions are female sex, younger age, smoking and ileocolonic CD.^84^ Finally, the TNF-α rs179996 allele has been identified as possible genetic risk factor for paradoxical psoriasis specifically in IBD patients.^190^

Whenever possible, discontinuation of anti-TNF should be avoided, as most cases respond well to topical dermatological treatments.^10^ In more severe cases, switching to another biologic (ustekinumab, vedolizumab, or secukinumab) can lead to partial or complete resolution, although ustekinumab and vedolizumab have been linked with IBD flares in a few patients.^177^^,^^182^^,^^191^ Infliximab-induced paradoxical psoriasis has also been treated successfully with guselkumab in an UC patient.^192^

### Herpes zoster

HZ is an infectious vesiculopapular skin disease (Figure 1) caused by the reactivation of varicella zoster virus (VZV), which remains dormant for years in sensory dorsal root or cranial nerve ganglia after initial exposure through chickenpox or VZV vaccination.^193^ It is important to note that ΗΖ is not transmissible from person to person; crusted lesions are not infectious, and contact with fluid from HZ blisters can only lead to primary varicella (chickenpox) in a naive individual or in cases of immunosuppression.^194^

Clinically, HZ presents as a vesiculopapular rash affecting 1 or more adjacent unilateral dermatomes, preceded by prodromal neuropathic symptoms such as pain, paresthesia, or dysesthesia (continuous or episodic) for 2 to 3 days before the rash appears. In immunocompetent patients, a few scattered lesions outside the affected dermatome may also occur, while immunocompromised patients may present with a widespread distribution (multiple dermatomes) of papulovesicular, bullous, or verrucous lesions that evolve beyond the typical 3- to 5-day time frame, lasting up to 2 weeks after initial presentation.^193^^,^^195^ Rarely, HZ may be symptomatic but without visible lesions, a condition called “zoster sine herpete.”^195^

IBD patients are at increased risk for HZ compared with the general population, with a meta-analysis^196^ reporting a 1.68 fold higher risk (incidence rate ratio [IRR], 1.68; 95% CI, 1.53-1.84; CD: IRR, 1.67; 95% CI, 1.40-1.98; UC: IRR, 1.49, 95% CI, 1.34-1.65). Known risk factors are increasing age (>50 years)/young age (<18 years), immunosuppression (especially impaired T cell immunity), active IBD/flares, Asian race, and history of HZ.^197^ This increased incidence has been linked to corticosteroids, thiopurines, and immunosuppressant/anti-TNF combination therapies, while data on anti-TNF monotherapy and methotrexate remain inconsistent.^197^^,^^198^ Although JAK inhibitors elevate the risk of HZ in other immune-mediated inflammatory diseases, a network meta-analysis found no significant difference compared with placebo in patients with IBD.^199^ Conversely, another meta-analysis identified a statistically significant association of HZ with tofacitinib (10 mg twice a day) and upadacitinib (45 mg every day), although JAK inhibitors as a class generally increase HZ risk.^200^ Among newer agents, including anti-IL-23, anti-integrins, anti-IL12/23, and anti-S1P, none demonstrated a statistically significant rise in HZ risk compared with placebo, with anti-IL-23 being the safest option.^200^

IBD is also associated with increased risk for all HZ complications except for ophthalmic ones; postherpetic neuralgia is the most frequent, followed by disseminated HZ and hospitalization.^201^ According to a recent large retrospective cohort study,^201^ risk factors for HZ complications are older age, comorbidities, and treatment with anti-TNF or prednisone, while non–anti-TNF, JAK inhibitors, and antivirals did not alter the risk. However, tofacitinib has been associated with a complicated course of HZ in previous studies.^197^

Diagnosis is mostly clinical. Regarding laboratory tests, polymerase chain reaction (PCR) for VZV DNA from active lesions is considered the gold standard, followed by direct fluorescent antibody to the VZV antigen.^193^ Crusted lesions should be deroofed and the sample for PCR should be taken from the base of the lesion.^197^ In disseminated HZ, quantitative PCR for VZV DNA in serum should be performed.^202^ No other tests are routinely recommended.^193^

Treatment consists of early initiation of systemic antivirals (<72 hours after first cutaneous lesions), wound care, and analgesics, although the latter do not influence postherpetic neuralgia risk.^193^ For immunocompetent patients without complications, the recommended treatment is oral valaciclovir 1000 mg 3 times a day for 7 to 10 days, while intravenous aciclovir (8-12 mg/kg body weight 3 times a day for 7 days) should be used in patients under immunosuppressives.^197^ In both cases, antivirals may be initiated after 72 hours if new lesions keep erupting after this window,^195^ and treatment for more than 7 to 10 days should be carefully considered in more severe cases.^203^

Vaccination for HZ can greatly reduce the risk of HZ, especially because the overwhelming majority of IBD patients with HZ are not vaccinated against it.^204^ According to the latest data on efficacy and safety of the recombinant zoster vaccine Shingrix, its efficacy in individuals >50  years of age remains high even 10 years postvaccination,^205^ making it a favorable option over live attenuated vaccines which are contraindicated in immunocompromised individuals.^197^ Furthermore, Shingrix may be administered in all high-risk immunosuppressed populations older than 18 years of age,^206^ although this practice has not yet been widely established. Therefore, it is reasonable to recommend that all IBD patients >50 years of age should get vaccinated against HZ, along with those considered high risk regardless of age.^197^

### Cutaneous EIMs and pregnancy

Pregnancy affects the course of IBD via hormonal alterations such as increased estrogen, progesterone, and human chorionic gonadotropin, which lead to increase of IL-10/other immunoregulatory cytokines and decrease of proinflammatory/inflammatory cytokines. Additionally, pregnancy can exacerbate intestinal dysbiosis.^207^ These changes may contribute to IBD flares, and hormonal imbalance increases the risk of EIMs.^208^ Furthermore, pregnancy is associated with various specific and nonspecific skin conditions, including polymorphic eruption of pregnancy, pemphigoid gestationis, atopic eruption of pregnancy, and pruritus due to intrahepatic cholestasis.^209^ Although these do not typically resemble cutaneous EIMs, their presence may further complicate the clinical picture, necessitating careful differentiation from IBD-related manifestations. Among the major shared dermatoses between IBD and pregnancy, EN is the most common, with a reported prevalence of 4.6% to 6% in childbearing women regardless of concurrent IBD.^210^^,^^211^ In contrast, PG is much rarer; Tan et al^212^ identified only 53 reported cases in the English-speaking literature, all of which were of the ulcerative phenotype, with 6 having concomitant UC.

When managing both IBD and EIMs during pregnancy, it is essential to prioritize the safety of both the mother and the fetus. Although anti-TNF agents are considered safe throughout pregnancy and breastfeeding.^213^ Huang et al^214^ suggested that they might increase the risk of abortions and preterm births. Of note, certolizumab does not cross the placenta.^215^ 5-Aminosalicylic acid, sulfasalazine, and thiopurines are considered generally safe, but initiation of thiopurines during pregnancy is not recommended due to maternal side effects and higher preeclampsia risk.^213^ Should antibiotics be used, penicillins are the safest option, while ciprofloxacin and metronidazole are best avoided during the first trimester and breast-feeding period. Corticosteroids may be used for short courses during IBD flares. MTX and thalidomide are absolutely contraindicated due to their teratogenic effects, while JAK inhibitors and ozanimod (S1P modulator) are not recommended due to limited data.

### Role of dermatologists

Dermatologists are essential in diagnosing and managing IBD-associated skin disorders, particularly severe conditions such as HS and PG. Their expertise ensures accurate diagnosis through clinical assessment and biopsies, allowing gastroenterologists to avoid unnecessary treatment modifications. Regular communication among specialists allows for timely adjustment of therapies, addressing any paradoxical reactions that may arise from biologic treatments. This coordination is especially critical in cases where biologics are used to manage both the gastrointestinal and cutaneous manifestations of IBD. Often, topical agents or other dermatological therapies can control skin disease, eliminating the need of systemic drug cessation. This approach not only improves treatment efficacy but also enhances the overall QoL for IBD patients.

In summary, dermatologists play a pivotal role in the diagnosis, treatment, and ongoing management of IBD-associated skin disorders, ensuring that patients receive the highest standard of care.

## Conclusions

Dermatological IBD manifestations are pleiotropic and encompass conditions directly linked to disease activity, those arising independently, and paradoxical reactions to biologics. Recognizing and appropriately managing these EIMs are paramount for improving patient outcomes and QoL. Advances in understanding the gut-skin axis, shared genetic predispositions, and immune dysregulation have opened avenues for novel therapies and personalized approaches. Interdisciplinary collaboration among gastroenterologists, dermatologists, and other specialists remains crucial in addressing the abovementioned complex conditions. As new treatments emerge, continuous research into their safety and efficacy will be pivotal in minimizing adverse effects and optimizing care for IBD patients with dermatological EIMs.

## Data Availability

No new data were generated or analysed in support of this research.
